# Family structure, parent-child conversation time and substance use among Chinese adolescents

**DOI:** 10.1186/1471-2458-10-503

**Published:** 2010-08-19

**Authors:** Kwok-Kei Mak, Sai-Yin Ho, G Neil Thomas, C Mary Schooling, Sarah M McGhee, Tai-Hing Lam

**Affiliations:** 1 School of Public Health, University of Hong Kong, Hong Kong; 2 Department of Public Health and Epidemiology, University of Birmingham, Birmingham, The UK

## Abstract

**Background:**

The family plays a vital role in shaping adolescent behaviours. The present study investigated the associations between family structure and substance use among Hong Kong Chinese adolescents.

**Methods:**

A total of 32,961 Form 1 to 5 (grade 7-12 in the US) Hong Kong students participated in the Youth Smoking Survey in 2003-4. An anonymous questionnaire was used to obtain information about family structure, daily duration of parent-child conversation, smoking, alcohol drinking and drug use. Logistic regression was used to calculate the adjusted odds ratios (OR) for each substance use by family structure.

**Results:**

Adjusting for sex, age, type of housing, parental smoking and school, adolescents from non-intact families were significantly more likely to be current smokers (OR = 1.62), weekly drinkers (OR = 1.72) and ever drug users (OR = 1.72), with significant linear increases in ORs from maternal, paternal to no-parent families compared with intact families. Furthermore, current smoking (OR = 1.41) and weekly drinking (OR = 1.46) were significantly more common among adolescents from paternal than maternal families. After adjusting for parent-child conversation time, the ORs for non-intact families remained significant compared with intact families, but the paternal-maternal differences were no longer significant.

**Conclusions:**

Non-intact families were associated with substance use among Hong Kong Chinese adolescents. The apparently stronger associations with substance use in paternal than maternal families were probably mediated by the poorer communication with the father.

## Background

Adolescence is regarded as the most challenging period in the life-course with many key biological, psychosocial and experiential changes all occurring within about 10 years [1]. How adolescents cope with these changes and find their way of life will have long-term influences on all aspects of their development including health. Substance use, including smoking, alcohol drinking and drug use, is one of the most important risk behaviours typically initiated during adolescence and carried over to adulthood. Recognising this, intensive public health research and interventions have been conducted to identify and control the risk factors of substance use, with some success in curbing consumption among adolescents in Western countries [2]. The World Health Organisation Framework Convention on Tobacco Control (FCTC) also calls for tighter public policies to restrict adolescent exposure and access to tobacco products [3]. However, as a risk factor of substance use, family structure has deteriorated and is not meant to be improved by the FCTC.

According to Jessor's Problem-Behaviour Theory [4], externalizing problems (smoking, alcohol drinking, and drug use) are predicted by the interaction between protective and risk factors in the family system. Strong family attachment with good parent-child communication may attenuate the adverse effect of non-intact family structure. The association between family structure and adolescent substance use was found to be mediated by maladjustment [5,6]. Life adjustment of children after parents' divorces depended on their communication with parents and custody in their later life [7]. Therefore, investigation on the relation between family structure and substance use should also consider parent-child communication.

Previous studies in Western populations have mostly found that adolescents from non-intact families were more likely to smoke, drink alcohol and take drugs [8-16] and to initiate drug use [17,18] compared with those from intact families. For instance, Adlaf et al (1996) reported that single-parent adolescents were 103%, 53% and 66% more likely to become heavy smokers, heavy drinkers and illicit drug users, respectively. However, contradictory findings have also been reported. While there were findings to support the association between non-intact family structure and higher alcohol drinking frequency among adolescents [19], others found no relation between them [20]. Relatively few studies have further classified single-parent families into paternal or maternal families [16,21-23], and none has made statistical comparisons between them in relation to adolescent substance use, and considering the effects of parent-child communication [24,25]. Most published studies on family structure and substance use are based on Western populations where the prevalence rates of non-intact families and adolescent substance use are both high [26]. Little is known about the associations in populations such as Asians where both non-intact families and substance use are relatively low. Apart from the differences in culture and parenting style [27-31], different perceived norms of family structure and substance use [32,33] may also affect the association between family structure and substance use. While the prevalence of substance use is similar between Western males and females, it is much less common in Asian women than men [34,35], which may contribute to paternal-maternal family differences in adolescent substance use.

Up to April 2006, the yearly divorce rate was 3.6 per 1,000 people in the United States [36] and the number of single mothers has increased from 3 to 10 million and single fathers from 393,000 to 2 million from 1970 to 2000 (U.S. Census Bureau). Although culturally, family bonding is typically strong among Asian populations, rapid economic development and westernization have resulted in major changes in values and lifestyles [37]. In China, the divorce rates had increased from 1.6 per 1000 in 1994 to 1.8 per 1000 in 2002 [38]. In Hong Kong, 81,644 children aged under 18 were living with single parents in 2001 and the number of such families had increased by 70% since 1991 [39]. With increasing numbers of Hong Kong people working in Mainland China, a rise of 132% from 98,300 in 1995 to 228,000 in 2005; family separation and divorces are likely to continue rising, and their effect on adolescent substance use is a cause for concern. Although slightly lower than those in the US, the divorce rates in Hong Kong had increased from 0.40 per 1,000 in 1981 to 2.54 per 1,000 in 2006 [40].

The objective of the present study was to investigate the association of four distinct family structures (intact, maternal, paternal and no-parent family) with smoking, alcohol drinking and drug use among an Asian population of Hong Kong Chinese adolescents, with a focus on paternal-maternal family differences and the role of parent-child communication.

## Methods

In the Hong Kong Youth Smoking Survey 2003-4, 85 secondary schools were randomly selected with a probability proportional to school enrollment size pursuant to the requirement of the Global Youth Tobacco Survey. All classes in Form 1 (grade 7 in the US) and two randomly selected classes each in Form 2 to 5 of the participating schools (in total 1012 classes) were surveyed.

A structured anonymous questionnaire was self-administered during normal school hours under the supervision of trained researchers and in the absence of teachers. The researchers explained the purpose of the study and emphasised that participation was totally voluntary. Completed questionnaires were sealed in an envelope immediately in the classroom and were sent to the University of Hong Kong directly. A total of 37,330 students participated in the survey. After excluding poorly answered questionnaires (response sets and missing information, etc), the number of valid responses reduced to 36,612 (98.1% of the original sample). Further excluding students with missing information on socio-demographic characteristics and family structure left 32,961 for the present analyses (88.3% of 37,330).

The schools sampled were similar to overall secondary schools in Hong Kong in the source of funding, district, and the proportion of coeducational schools. In line with local practice for general school surveys, passive consent was sought from parents emphasizing that participation was totally voluntary. Ethical approval for the study was granted by the Institutional Review Board of the University of Hong Kong/Hospital Authority Hong Kong West Cluster.

"Whether a parent is present" and "parent-child conversation time" were asked using the item "How long daily on average do you converse with you father/mother (in separate items)". Eight options were provided, including "no father/mother", "no conversation at all" and 6 options of increasing durations (from " < 1 min" to "30 min or above") on an average day. The first option of "no father/mother" indicated the absence, and all other options indicated the presence of the father or mother. Family structure was then classified into intact family (with both parents) and non-intact family including maternal, paternal and no-parent families. The method of parent-child conversation was not specified in the questionnaire so that the students were expected to include both face to face and telephone conversations. Parent-child conversation was used as a proxy of the quality of parent-child communication. Although longer duration of conversation does not necessarily mean better parent-child communication, it has the advantage of being a simple and factual measure.

Smoking was analysed as current smoking (any smoking in the past 30 days) versus non-current smoking. Alcohol drinking was classified as weekly or more frequent versus less than weekly. Drug use referred to psychotropic substances such as cannabis, ecstasy and ketamine, but excluding drugs prescribed by doctors. Since adolescent drug use was uncommon, its usage was classified as ever versus never. To control for the potential influence of parental substance use, parental smoking was assessed using the item "Do your parents smoke?" with 5 options of "both do not smoke", "both smoke", "only father smokes", "only mother smokes" and "don't know". Regardless of family structure, most students were able to report the smoking status of both parents. We did not collect information on parental drinking and drug use in the present survey.

Binary logistic regression was used to calculate the adjusted odds ratios (ORs) for the use of each substance by family structure in 2 models. Model A adjusted for sex, age, type of housing and parental smoking; model B additionally adjusted for parent-child conversation time to test the independent effect of family structure. For single-parent families, the conversation time with the only parent was used. For intact families, the longer conversation time between the two parents was used. The sum of the paternal and maternal conversation time was not used because some of these conversations might have occurred at the same time and their effects may not be additive. Model B was not applicable to no-parent families because by definition these families had no parent-child conversation. In a sensitivity analysis, similar results were obtained by using the mean conversation time with the father and mother for intact families.

Adjusted odds ratios were calculated for (1) non-intact versus intact families, (2) maternal, paternal and no-parent versus intact families with tests for linear trend, and (3) paternal versus maternal families. Potential moderating effects of sex and age on family structure were examined by including respective interaction terms in each regression model. The potential clustering effects of school were controlled by robust estimators in all regression models.

## Results

Among 32,961 students, 6.7% were from non-intact families, including maternal (4.8%), paternal (1.1%) and no-parent (0.8%) families. Table 1 shows that boys were over-represented in no-parent (68.1%) and paternal (55.0%) families but under-represented in maternal families (42.8%) compared with their overall proportion of 46.7%. The greater proportion of students from maternal families than paternal families, and the greater proportion of girls than boys among single-parent families, were both consistent with local census findings [41]. As a proxy for higher socioeconomic status, private housing was most prevalent among intact families (51.0%), followed by paternal (45.6%), maternal (39.5%) and no-parent families (22.4%). Any parental smoking was more common in non-intact families, and fathers were more likely than mothers to smoke regardless of family structure. Adolescent substance use progressively increased from intact to maternal, paternal and no-parent families consistently for current smoking (7.9% to 18.9%), weekly drinking (6.1% to 21.6%) and ever drug use (6.0% to 17.8%) alike.

**Table 1:** Characteristics of respondents by family structure

	Intact (n = 30749)	Maternal (n = 1598)	Paternal (n = 360)	No-parent (n = 254)	Total (n = 32961)	*Non-intact (n = 2212)
	**n (%)**	**n (%)**	**n (%)**	**n (%)**	**n (%)**	**n (%)**

Sex						
Boys	14354 (46.7)	684 (42.8)	198 (55.0)	173 (68.1)	15409 (46.7)	1055 (47.7)
Girls	16395 (53.5)	914 (57.2)	162 (45.0)	81 (31.9)	17552 (53.3)	1157 (52.3)
						
Age group						
12 or below	7066 (23.0)	296 (18.5)	77 (21.4)	58 (22.8)	7497 (22.7)	431 (19.5)
13	5485 (17.8)	226 (14.1)	63 (17.5)	60 (23.6)	5834 (17.7)	349 (15.8)
14	4860 (15.8)	244 (15.3)	61 (16.9)	30 (11.8)	5195 (15.8)	335 (15.1)
15	4061 (16.1)	292 (18.3)	48 (13.3)	33 (13.0)	5334 (16.2)	373 (16.9)
16	5066 (16.5)	288 (18.0)	70 (19.4)	45 (17.7)	5469 (16.6)	403 (18.2)
17 or above	3311 (10.8)	252 (15.8)	41 (11.4)	28 (11.0)	3632 (11.0)	321 (14.5)
						
Housing type						
Private/subsidized	15578 (51.0)	622 (39.5)	162 (45.6)	56 (22.4)	16418 (50.1)	840 (38.5)
Public	12990 (42.5)	858 (54.4)	157 (44.2)	162 (64.8)	14167 (43.3)	1177 (54.0)
Temporary or others	1993 (6.5)	96 (6.1)	36 (10.1)	32 (12.8)	2157 (6.6)	164 (7.5)
						
Parental Smoking						
Both smoke	17630 (57.4)	833 (52.2)	164 (45.6)	112 (44.4)	17630 (57.4)	11.9 (50.2)
Only father	11089 (36.1)	522 (32.7)	151 (41.9)	93 (36.9)	11089 (36.1)	766 (34.7)
Only mother	340 (1.1)	61 (3.8)	7 (1.9)	7 (2.8)	340 (1.1)	75 (3.4)
Both do not	1125 (3.7)	98 (6.1)	25 (6.9)	19 (7.5)	1125 (3.7)	142 (6.4)
Don't know	535 (1.7)	82 (5.1)	13 (3.6)	21 (8.3)	535 (1.7)	116 (5.3)
						
Smoking						
Non-current	28296 (92.1)	1409 (88.2)	300 (83.3)	206 (81.1)	30211 (91.7)	1915 (86.6)
Current	2437 (7.9)	188 (11.8)	60 (16.7)	48 (18.9)	2733 (8.3)	296 (13.4)
						
Alcohol drinking						
Less than weekly	28783 (93.9)	1465 (92.0)	312 (87.4)	196 (78.4)	30756 (93.6)	1973 (89.7)
Weekly	1880 (6.1)	128 (8.0)	45 (12.6)	54 (21.6)	2107 (6.4)	227 (10.3)
						
Drug use						
Never	28837 (94.0)	1455 (91.2)	316 (88.3)	208 (82.2)	30816 (93.7)	1979 (89.7)
Ever	1835 (6.0)	140 (8.8)	42 (11.7)	45 (17.8)	2062 (6.3)	227 (10.3)

*Includes paternal, maternal or no-parent family

In Table 2, intact families generally had longer father-child and mother-child conversation time than single-parent families. No conversation at all and short conversation time were more common in non-intact families, and conversation for over 30 minutes was more common among the mothers (40.7%) and fathers (21.3%) in intact families.

**Table 2:** Parent-child conversation time by family structure

	Intact (n = 30749)	Maternal (n = 1598)	Paternal (n = 360)
	
	n (%)	n (%)	n (%)
Average conversation time (min) with mother/day			
None	4.1	7.4	-
< 1	4.5	6.1	-
1-4	9.4	13.6	-
5-9	11.9	12.0	-
10-19	16.0	16.9	-
20-29	13.4	10.7	-
30 or above	40.7	33.2	-
			
Average conversation time (min) with father/day			
None	9.3	-	19.8
< 1	12.0	-	13.6
1-4	17.4	-	18.7
5-9	15.3	-	11.0
10-19	15.8	-	15.3
20-29	9.0	-	7.1
30 or above	21.3	-	14.7

Model A in Table 3 shows highly significant ORs that generally increased from intact to maternal, paternal and no-parent families (p < 0.001 for trend). Overall, adolescents from non-intact families were 62%, 72% and 72% more likely to be current smokers, weekly drinkers and ever drug users, respectively, compared with intact families (all p < 0.001). Further adjusting for parent-child conversation time in Model B reduced the ORs, but they remained statistically significant. In Table 4, model A shows that compared with maternal families, adolescents from paternal families were significantly more likely to be current smokers and weekly drinkers with ORs (95%CI) of 1.41 (1.02-1.95) and 1.46 (1.01-2.12), respectively. However, none of the paternal-maternal differences in substance use was significant with further adjustment of parent-child conversation time. None of the above associations between family structure and substance use was modified by sex or age.

**Table 3:** Adjusted* odds ratios for adolescent substance use in non-intact families compared with intact families

		Intact	Maternal		Paternal		No-parent			Non-intact vs intact	
		
		OR	OR (95% CI)	P	OR (95% CI)	P	OR (95% CI)	P	P for trend	OR (95% CI)	P
	Model										

Smoking											
Current vs non-current	A	1	1.44 (1.22-1.69)	< 0.001	2.09 (1.57-2.80)	< 0.001	2.09 (1.49-2.94)	< 0.001	< 0.001	1.62 (1.42-1.85)	< 0.001
	B	1	1.27 (1.07-1.50)	0.005	1.55 (1.15-2.08)	0.004	/	/	/	1.20 (1.04-1.38)	0.013
Alcohol drinking											
Weekly vs less than weekly	A	1	1.37 (1.13-1.66)	0.001	1.97 (1.42-2.72)	< 0.001	3.71 (2.70-5.10)	< 0.001	< 0.001	1.72 (1.48-2.00)	< 0.001
	B	1	1.27 (1.05-1.54)	0.015	1.69 (1.21-2.34)	0.002	/	/	/	1.43 (1.22-1.67)	< 0.001
Drug use											
Ever vs never	A	1	1.47 (1.22-1.76)	< 0.001	1.96 (1.41-2.73)	< 0.001	3.05 (2.18-4.26)	< 0.001	< 0.001	1.72 (1.48-2.00)	< 0.001
	B	1	1.40 (1.17-1.69)	< 0.001	1.67 (1.20-2.33)	0.002	/	/	/	1.47 (1.26-1.72)	< 0.001
											

*Model A adjusted for sex, age, housing type, parental smoking, and school effect; Model B additionally adjusted for parent-child conversation time

**Table 4:** Adjusted* odds ratios for adolescent substance use in paternal compared with maternal families

	Model	OR (95% CI)	P
Smoking			
Current vs non-current	A	1.41 (1.02-1.95)	0.039
	B	1.27 (0.91-1.78)	0.16
Alcohol drinking			
Weekly vs less than weekly	A	1.46 (1.01-2.12)	0.046
	B	1.28 (0.87-1.88)	0.22
Drug use			
Ever vs never	A	1.31 (0.90-1.91)	0.15
	B	1.19 (0.81-1.75)	0.37

*Model A adjusted for sex, age, housing type, parental smoking, and school effect; Model B additionally adjusted for parent-child conversation time

## Discussion

Consistent with previous studies, we found that adolescents from non-intact families were more likely to be current smokers, weekly drinkers and ever drug users. Furthermore, by adjusting for parental smoking, we provided evidence that the association between family structure and adolescent smoking was not due to confounding by higher parental smoking rates among non-intact families. Although parental drinking and drug use were not recorded, their effects have probably been partially accounted for by the adjustment of parental smoking due to its correlation with drinking [42] and drug use [43]. The ORs for substance use were attenuated after adjusting for parent-child conversation time, suggesting that the associations were partially explained by parent-child communication.

The apparently stronger associations of adolescent substance use with paternal than maternal families could be attributed to several factors. Single fathers (71.0%) are more likely than single mothers (52.8%) to work rather than to stay at home taking care of the children [41]. Furthermore, mothers tend to know more about the daily activities of their children [44], and are more likely to advise on health issues such as the harm of smoking [45]. Such paternal-maternal differences become statistically insignificant after adjusting for parent-child conversation time, suggesting that the effects of paternal families were mediated through poorer communication between the child and the father.

Paternal-maternal differences in socioeconomic status, as reflected by the type of housing, were small and adjusted for in the analysis. Comprehensive Social Security Assistance is also available for families with financial difficulties. Therefore socioeconomic status was unlikely to be a main factor for the paternal-maternal differences observed. Some studies have reported that the effect of family structure on substance use differed significantly by sex [20,46], but this was not observed in the present study.

Our study has the advantage of having a large and representative sample, but it also has several limitations. The schools were sampled from mainstream secondary schools only, and international schools, constituting 4.4% of all secondary schools [47] were excluded. Form one students were purposely over-sampled and there were slightly more girls than expected in the sample. These might have slightly affected the prevalence of family types and overall substance use, but were unlikely to have much influence on the associations investigated. The prevalence of single-parent families in our study (5.9%) was also similar to that reported in another study (5.5%) among Hong Kong adolescents [48], which supported the validity of our data.

Family structure was derived from two questions on the daily duration of conversation with the father and the mother rather than asking the students directly about the marital status of their parents. Most likely, the absence of a parent would be due to marital separation or divorce rather than death as most parents should be at their 40s only and life expectancy in Hong Kong is among the highest worldwide. On the other hand, it was possible that a parent considered to be present but with no conversation with the child was actually maritally separated from the family. In a sensitivity analysis, the category of "no conversation at all" was also treated as absence of the parent. This indeed produced larger ORs for non-intact families overall and paternal families in particular, resulting in significant differences between paternal and maternal families for all measures of substance use even with adjustment of parent-child conversation time. We have also included other potential confounders such as exercise frequency and participation in volunteer work in our analyses, but they had little effect on the risk estimates.

We have only quantitatively measured the conversation time, without taking into account the quality of conversation, which may also be important to the prevention of substance use in adolescents [49]. Within the quality dimension, openness of both parties [50] as well as contexts of the conversation may also be important factors associated with adolescent substance use [51]. In addition, perceived support from parents [52] rather than the actual conversation, and different family activity time other than talking [53], may also enhance resilience to substance use in adolescents. We have included parental smoking which may potentially confound the association between family structure and substance use [54,55] in our analyses. Socioeconomic status was only assessed using a crude measure of the type of housing, and other characteristics such as parental occupation and income were not considered. Peer-influence on adolescent substance use was also not assessed. These factors should be considered in future studies. Due to the cross-sectional nature of the survey and the lack of detailed information about marital status of the parents, the temporal sequence between family structure and substance use cannot be ascertained. However, both smoking and alcohol drinking referred to current consumption while family structure could have been formed much earlier and remained stable for most respondents.

Our findings should be particularly relevant to Asian populations in which large number of families are split, in particular, due to rural women seeking urban employment [56] or overseas employment as domestic helpers, many leaving their children with their father. Rapid economic development in this region and globalisation will likely make the situation worse in the future. Few other risk factors may affect adolescent development and health as extensively as family structure does, but ironically it also makes family structure "no-man's-land" in public health intervention. Better family-friendly public policies for local employment and work arrangements are needed to help families maintain their intactness. As family unity is highly regarded in Chinese societies, the lack of such in a small minority of families in Hong Kong may have larger impact on adolescent health than in Western countries where individualism and independence are valued and non-intact families are common. Due to their relative rarity, adolescents from non-intact families in Hong Kong may also have less social support from peers with similar backgrounds compared with their Western counterparts. Schools may have an important role in helping students from non-intact families, and innovative interventions may be required to reach single-fathers who are reluctant to seek help.

## Conclusions

Non-intact families were associated with substance use among Hong Kong Chinese adolescents. The apparently stronger associations with substance use in paternal than maternal families were probably mediated by the poorer communication with the father.

## Competing interests

The authors declare that they have no competing interests.

## Authors' contributions

KKM performed statistical analyses, interpretation of data, drafted and revised the manuscript; SYH is the principle investigator of the HKYSS project and critically revised the manuscript; GNT, CMS, and SMM revised the manuscript for important intellectual content; THL contributed to conception and design of the study, and revised the manuscript; All authors read and approved the final manuscript.

## Pre-publication history

The pre-publication history for this paper can be accessed here:

http://www.biomedcentral.com/1471-2458/10/503/prepub
